# Voices behind the Statistics: A Systematic Literature Review of the Lived Experience of Rheumatic Heart Disease

**DOI:** 10.3390/ijerph17041347

**Published:** 2020-02-19

**Authors:** Emma Haynes, Alice Mitchell, Stephanie Enkel, Rosemary Wyber, Dawn Bessarab

**Affiliations:** 1 School of Population and Global Health, The University of Western Australia, Perth 6000, Australia; 2 Telethon Kids Institute, Perth 6000, Australia; stephanie.enkel@telethonkids.org.au (S.E.); rosemary.wyber@telethonkids.org.au (R.W.); 3 Menzies School of Health Research, Charles Darwin University, Darwin 0810, Australia; alice.mitchell@menzies.edu.au; 4 The George Institute for Global Health, University of New South Wales, Sydney 2000, Australia; 5 Centre for Aboriginal Medical and Dental Health, The University of Western Australia, Perth 6000, Australia; dawn.bessarab@uwa.edu.au

**Keywords:** rheumatic heart disease, lived experience, Indigenous, collective trauma, adolescent, power, strengths-based

## Abstract

In Australia, Aboriginal children almost entirely bear the burden of acute rheumatic fever (ARF) which often leads to rheumatic heart disease (RHD), a significant marker of inequity in Indigenous and non-Indigenous health experiences. Efforts to eradicate RHD have been unsuccessful partly due to lack of attention to voices, opinions and understandings of the people behind the statistics. This systematic review presents a critical, interpretive analysis of publications that include lived experiences of RHD. The review approach was strengths-based, informed by privileging Indigenous knowledges, perspectives and experiences, and drawing on Postcolonialism and Critical Race Theory. Fifteen publications were analysed. Nine themes were organised into three domains which interact synergistically: sociological, disease specific and health service factors. A secondary sociolinguistic analysis of quotes within the publications articulated the combined impact of these factors as ‘collective trauma’. Paucity of qualitative literature and a strong biomedical focus in the dominant narratives regarding RHD limited the findings from the reviewed publications. Noteworthy omissions included: experiences of children/adolescents; evidence of Indigenous priorities and perspectives for healthcare; discussions of power; recognition of the centrality of Indigenous knowledges and strengths; and lack of critical reflection on impacts of a dominant biomedical approach to healthcare. Privileging a biomedical approach alone is to continue colonising Indigenous healthcare.

## 1. Introduction

Indigenous Australians are the oldest continuous civilisation in the world [1], occupying the continent for at least 45–65,000 years. During this period, complex and varied knowledge systems embedded in culture, language and law were developed [2]. Over the last 200 years, an ongoing process of colonisation has assailed these strengths. The impacts of colonisation are starkly demonstrated in statistical comparisons of Indigenous and non-Indigenous health. Within a raft of ‘gaps’ in health status measures, rheumatic heart disease (RHD) stands out as an exemplar of the differences between Indigenous and non-Indigenous health experiences.

Acute rheumatic fever (ARF) is an autoimmune disease occurring some weeks after an untreated Group A streptococcus (GAS) infection of the throat or skin [3]. Severe ARF and/or ARF recurrences can lead to RHD, involving permanent damage to the heart valves leading to ongoing disability and premature death. Currently, there is no cure for RHD and treatment following an episode of ARF requires a painful injection of long-acting penicillin every 21–28 days for at least a decade to prevent ARF recurrences and progression to RHD [4,5]. Approximately 420 cases of ARF are diagnosed among Australian Aboriginal and Torres Strait Islander people annually, [6] a rate 123 times higher than rates in non-Indigenous people [7]. Further, of the more than 5000 people living with RHD in Australia, 71% are Aboriginal and Torres Strait Islander people [7]. This represents one of the highest burdens of RHD in the world. The consequences of ARF and RHD are enormous, significantly impacting, including shortening, the lives of Aboriginal and Torres Strait Islander children, adolescents, young adults, individuals, families and communities in Australia [8,9,10]. 

Despite the glaring evidence of disparity described by the statistics related to RHD, long-term concerted efforts to eradicate RHD have had little success [11]. Arguably, part of the problem is a lack of attention to the voices, opinions and understandings of the people behind these statistics. The use of statistical comparisons between Indigenous and non-Indigenous populations is innately Othering and is *“unlikely to deviate from well-worn themes of disadvantage and deviation from the norm”* [12], with an *“inherent potential to underpin pejorative discourses of Indigenous lived reality”* [12]. This leads to problematising Indigenous health without considering either Indigenous peoples’ strengths or how the impacts of colonisation, and the dominance of biomedical discourses continue to influence the ongoing health narrative of Aboriginal people.

Healthcare strategies formulated solely on non-Indigenous knowledge are unlikely to achieve a lessening of health disparities for Aboriginal people and may even lead to a widening of the gap [13]. It is therefore encouraging that, given the non-medical effects of RHD have been underexplored, greater attention to the lived experience of children, adults and communities with RHD has recently been prioritised by the RHD clinical and research community [3]. In order to improve health service delivery based on Indigenous knowledges, it is hoped that a better understanding of the lived realities of RHD might provide insight into ways to empower Aboriginal children, families and communities and improve their health and wellbeing. 

The overall purpose of the systematic review is knowledge building/theory generating, employing the analysis methods described by Finfgeld-Connett and Johnson [14]. This article describes the processes and findings from a systematic review and a critical, interpretive analysis of publications that include information on the lived experiences of ARF and RHD. In particular, the review seeks, where possible, to prioritise the voice of Indigenous participants within the reviewed publications.

## 2. Theoretical Approach 

This article aims to have a strengths-based research approach, informed by privileging Indigenous knowledges, perspectives and experiences, and drawing on Postcolonialism as a theoretical tool and Critical Race Theory. 

### 2.1. Postcolonialism 

The processes of colonialism involve essentialising a set of characteristics to create an Other that can then be judged against the Western ideal [15]. This process empowers those embedded in Western culture to acquire and maintain a strong sense of identity by contrasting themselves with the Other. Othering effectively marginalises and suppresses Indigenous knowledge systems and ways of knowing [16]. A systemic impact of colonialism upon Indigenous Australian’s health is that Indigenous people are continually positioned as the problem [17]. Postcolonialism seeks to decolonise, to disrupt dominant Western views by privileging the Indigenous voices previously silenced by dominant ideologies [18].

### 2.2. Critical Race Theory 

In addition to privileging Indigenous knowledges, opinions and experiences, Critical Race Theory requires the contextualising of experiences within relations of power. Failing to do so *“enables avoidance of dealing with the causal agents and sustains an Indigenous problem-based approach”* [19]. Deconstructing and unpacking the structural conditions of disadvantage and focusing on power relations allows present realities to be understood within the context of the history and legacy of colonisation and its impacts on Indigenous health [20].

## 3. Methods

Initially, the aim of this study was to explore the evidence from publications reporting on the lived experience of people and communities at risk of and living with ARF and RHD in Australia and New Zealand. As described in more detail below, during the review process the decision was made to only analyse in depth the Australian publications. The following sections describe, the data selection and data analysis methods. 

### 3.1. Data Selection 

A systematic search of academic and grey literature was undertaken between March and April 2019. Databases searched included Web of Science, Cinahl Plus, Embase, PubMed, Medline, Cochrane, Global Health and JBI COnNECT. Search terms used were (‘rheumatic fever’ OR ‘rheumatic heart disease’) AND (‘Australia’ OR ‘New Zealand’) as well as an expansive collection of terms to specifically capture qualitative research as a proxy search term for lived experience (Appendix A.1). Strategies were adapted to suit the unique requirements of each database prior to searching. Google Scholar was searched specifically for grey literature using a shortened search strategy (Appendix A.1) and a number of government databases were accessed for additional results. 

Several limitations were noted in the review. There was only a small body of Australian literature investigating the research question, the retrieval of qualitative research is challenging given such factors as limited indexing, non-indicative titles and abstracts [21], and there is a predominance of quantitative studies in this field. Thus, while Australia was the primary country of focus, the New Zealand literature was initially included for exploration due to similarities in population demographics, colonisation experience, models of care and current research undertakings in order to augment the body of evidence for review.

Of the 915 results returned across these search methods, 909 were able to be extracted. After the removal of duplicates, 528 were assessed for inclusion. Papers were selected for analysis if they explored Indigenous participants’ ARF/RHD experiences, were conducted qualitatively or if they were mixed methods studies that reported qualitative findings. Conventional systematic review inclusion criteria were broadened to include publications that might otherwise have been omitted (such as editorials and case studies). In theory, this allowed for greater inclusion of publications authored by an Indigenous person or including Indigenous people as co-researchers. 

**Figure 1:**
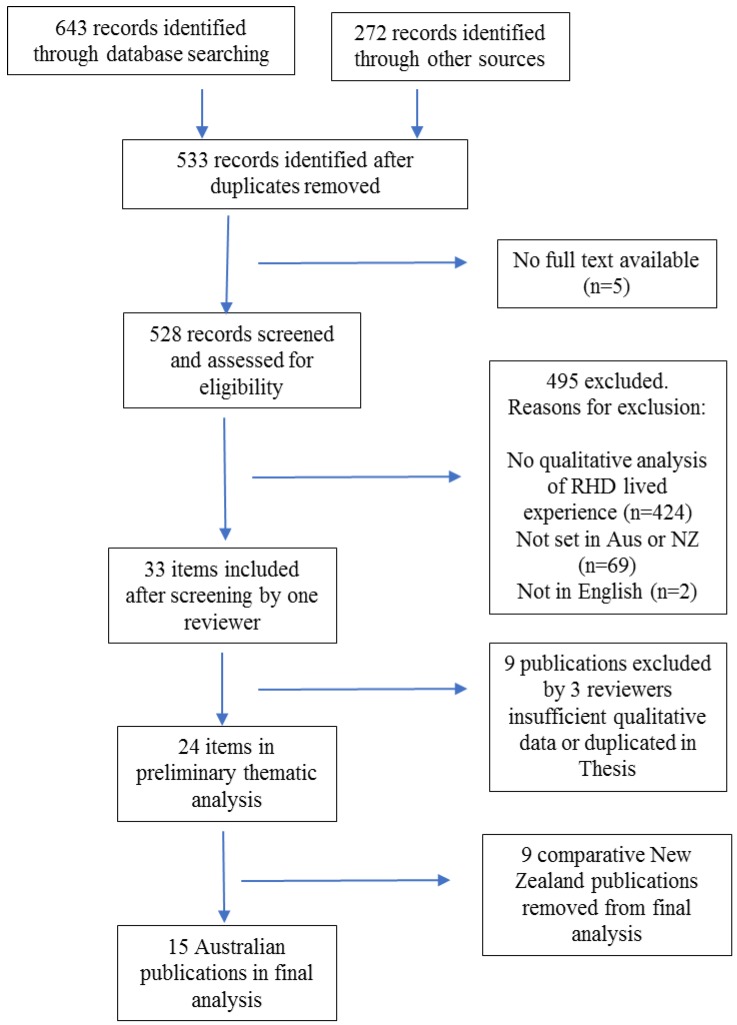
Literature search flow diagram.

After the application of these criteria, 24 publications were identified. In the initial analysis process, despite considerable thematic similarities, significant differences were noted in healthcare delivery between Australia and New Zealand which impacted differently on lived experience. Therefore, we primarily report here on the 15 Australian publications, and only include findings from the New Zealand literature where they are notably different or, conversely, add depth to themes from the Australian literature. We found that only one sub-theme relating to medication costs was specific to New Zealand. This process is demonstrated in Figure 1.

Four co-authors participated in assessing each publication using a hierarchy of qualitative research evidence [22] (Appendix A.2). No papers were excluded as a result of the quality appraisal, rather in the Discussion the hierarchy level was used to comment on the strength of findings from the thematic analysis. 

### 3.2. Data Analysis

Using a critical decolonising lens, review authors (including one senior Indigenous research leader and two non-Indigenous researchers with extensive experience in Indigenous health) contributed to an inductive thematic analysis of the lived experience evidence from all publications. An inductive, narrative content analysis method was used to develop themes from the reviewed literature. The narrative analysis identified and summarised key ideas within the full text of the reviewed publications. These key ideas were then coded inductively (meaning that initial codes were developed from the data without prior assumptions) to construct the themes. This analysis method aligns with that described by Finfgeld-Connett and Johnson [14]. The commonalities and relationships between the concepts are identified in order to make generalisations that are intended to enhance research, practice and policy formation [14]. This method promotes the development of critical analysis and aims to provide a synthesis that moves beyond the reported themes from the primary studies [23] (see Discussion).

Specifically, four of the authors created a coding template by first doing a full text review of all included publications (each author coding a quarter of the publications) to identify dominant themes. The template that was then used to complete a detailed coding of allocated publications, adding themes and sub-themes as they arose. Co-authors met frequently to discuss the initial themes and template, new themes and any individual coding anomalies/issues, thereby iteratively developing, refining and validating themes and sub-themes. In all discussions, the input and expertise of the Indigenous researcher was central.

Finally, the quality of all reviewed publications was assessed according to the established criteria for assessing evidence from qualitative research for clinical decision-making [22]. This hierarchy of evidence allows determination of the reliability/generalisability of the findings from the reviewed publications to inform policy and practice. Thus level 1 provides the highest *“transferable evidence that is generalisable beyond the setting where the study is conducted”* [22] (Details of criteria are shown in Appendix A.2). The final set of themes and sub-themes was then combined with the hierarchy of evidence ([22]) to create a coding matrix that enabled comparative analysis of the frequency and strength of sub-themes for each article (Table 2 and Appendix A.3).

Additionally, reflecting cultural safety principles of prioritising direct Indigenous voices over authors’ synthesis [24], a sociolinguistic analysis was undertaken of the combined research participants’ quotes extracted from all publications. This method addresses concerns *“that participants’ voices become diluted through the sequential filters of selection, interview, analysis, publication and meta-synthesis”* [24], particularly when academic authors are drawing conclusions from disenfranchised voices.

## 4. Results

In all, 15 publications were identified for review. All were published during the past 16 years with the majority (10) published since 2016. The sociolinguistic analysis of combined research participants’ quotes extracted from all publications included 201 quotes estimated to represent at least 129 individuals. Rural and/or remote areas were the primary setting of research in all but two instances (Table 1).

The majority (11) of included research were peer-reviewed publications extracted from academic journals. Of these, most were published in domestic biomedical journals. Two included publications were theses (one Masters level research and one PhD), and there was one report. Half the publications (7) were assessed to be Level 1 publications according to the hierarchy of evidence [22], providing strong, generalisable evidence. The publications that either intentionally or indirectly explored lived experience were evenly spread across the hierarchy of evidence with only two considered to be at Level 1, two other publications were considered to be Level 2 of the hierarchy, and one was identified as Level 4. The inclusion of an Indigenous author was identified in more than half (8) of the publications. Investigating barriers to the uptake of secondary prophylaxis was the most frequent study aim (6), with others focusing on the ARF/RHD journey in its entirety (4) (including disease understanding, awareness and health literacy), RHD in pregnancy (2) and transition to adult care (1).

Authors’ disciplines were recorded due to qualitative research methods being increasingly used by researchers trained in quantitative methods. Publications were predominantly from within medical or clinical science disciplines (six), with only three considered ‘social science’ publications (anthropological, linguistic or sociological), and six publications demonstrating a collaboration between biomedical and social researchers in the undertaking of the investigation.

Eight of the 15 Australian publications were driven by pragmatic research goals—to address functional, managerial or evaluation questions, such as how to ensure patients stayed on target with secondary prophylaxis by assessing barriers and enablers of care, evaluating interventions aiming to better manage RHD or measuring service planning, program development and cost output. The remaining seven publications either intentionally or indirectly explored the lived experiences of ARF and RHD independently of other research agendas. 

### 4.1. Thematic Analysis Findings

The nine themes identified during the analysis have been organised into three domains: sociological (socioeconomic, political, historical and cultural); disease specific (the immediate health and logistical impacts of Strep A infections, ARF and RHD); and health service factors. However, while described separately, it is clear that all domains concurrently impact on lived experience. The results of the sociolinguistic analysis of quotes from Indigenous people articulate the combined impact of these factors as ‘collective trauma’. The nine primary themes analysed according to frequency and hierarchy level are recorded in Table 2 (further detail including the 27 sub-themes identified within the 9 themes, can be accessed in Appendix A.3).

#### 4.1.1. Sociological Domain 

Some of the reviewed publications described RHD using the phrase *“a disease of poverty”* [8,9,25,31,32,33] as is common in biomedical papers. Analysis revealed two themes (discussed below) that demonstrate the extent to which sociological factors, including experiences related to racism, powerlessness and poverty, are a major determinant of the ‘lived experience’ of disease. Finally, to disrupt the tendency to problematise Indigenous health, our analysis identified publications that recognised or described social and cultural strengths that have positive impacts on the health of people with RHD/ARF. This is the third theme in this domain.

##### Lived Realities of Children, Adults and Families Affected by RHD

The majority of publications identified sociological factors impacting on people affected by RHD. Poverty was described as a significant barrier to making health-enhancing choices, including food choices [8,9,27,28]. As stated in Gruen, there is no *“escape from the hardship of daily existence and few opportunities to improve their situation”* [24] in settings of poverty.

*“No car to go hunting, an intermittent supply of food, no phone, no money for a bus fare were all mentioned as impacting either on the patient’s wellbeing, or on their ability to access medical services” Harrington, 2005* [27]

The reviewed publications described stresses related to a lack of transport, particularly when there are frequent requirements to travel for health care [8,9,25,26,29,36].

Having a large number of people in the house was described as causing hunger [9,25], and was equated with children getting sick, and feeling judged or *“spied on”* by healthcare providers for not meeting Western expectations in house standards [27]. Greater emphasis was placed on the impacts of housing on lived experience in the New Zealand literature and included impacts of family issues, parents being incarcerated, children and adolescents being in foster care and needing to draw on whānau (Maori extended family/community of families) support [37,38,39].

A feature of entrenched disadvantage is the experience of multiple intersecting practical challenges, both individual and collective, often resulting in conflicting priorities. The reviewed literature described the intersection of inequities (such as the impact of poverty, indifference born out of lack of opportunities, social and cultural obligations, gender sensitivities, domestic violence, language and communication difficulties and service access issues) as factors adding to the complexities of living with RHD [8,9,25,35].

##### Experiences of Power Differences and Racism

The overall collective voices in the publications show that connecting with primary healthcare is fraught with experiences of racism and powerlessness. Ongoing colonisation and the impact of systemic racism were described as contributing to poor experiences of RHD and difficulties in accessing primary healthcare [8,10,25,28,32,33]. In essence, those living with ARF and RHD are doing so in the midst of a system mismatched to their needs, and thus ‘compromising care’ [33]. The dominant culture background and biomedical worldview of healthcare providers contributes to the power imbalance [9,10,25,27,30,32,33,35] with the result that participants felt “*alienated by the very services intended to care for them*” [34] and a lack of trust, *“they tell us a lying story”* [30].

The perception, held by healthcare providers, individuals, families and communities regarding the inevitability of illness and skin sores, contributes to care being neither sought nor offered, and a sense of powerlessness for individuals and families [8,9,25,28,30,33]. This was so profound that normalisation seemed to act almost as a determinant of health in its own right.

“You can’t stop RHD”; “Yeah yeah. Like you can get that from generation to generation, passing it down. It’s in our family.” Pregnant Aboriginal women quoted in Belton et al., 2016 [9]

The New Zealand literature gave more depth to descriptions of the impacts of power differentials, describing a lack of trust, feeling judged, having to ‘push’ to have throat swabs taken and diagnostic failures even when family members suspected ARF [37,40,41].

##### Indigenous Culture, Knowledge and Strengths

In nearly half of the reviewed publications descriptions of protective factors associated with Indigenous ways of being and doing were found. Whilst cultural obligations add to the many competing demands of daily life, they also provide positive experiences [26,27,28] and living in communities can strengthen this connection to culture [9,25,26,27,28,30]. Despite the tendency to problematise Indigenous health half (7/15) of the reviewed publications included descriptions of protective factors of Indigenous ways of being and doing [9,25,26,27,28,30,35]. These social and cultural strengths within Indigenous groups may mitigate some of the difficulties of living with RHD (see also sub-theme: Factors contributing to positive experiences of health-care).

*“I don’t feel sick back home. Yeah, because of that bush medicine. Yeah, and I got my grandmother like, cook for a person when they’re sick. With the medicine from the tree, yeah.’ Pregnant Aboriginal women quoted in Belton et al., 2016* [9]

These strengths, as described above, are culturally mandated and connect to local knowledge and languages. They are also relationship-based and include ways of caring for each other and the strengths gained from being cemented as part of a group [9,25,27,28,35].

#### 4.1.2. Disease Specific Domain 

Many factors associated with RHD are common to other health experiences (for example, poor communication, inadequate access to primary healthcare, lack of appropriate information). The reviewed publications also reflected specific issues associated with RHD. 

##### Disease-Specific and Traumatic Impacts of RHD

The condition-specific complexities of RHD include the preceding infection, acute and chronic phases. For patients, this can mean adhering to a long term, often painful, secondary prophylaxis regimen; suffering restrictions on normal activities; travelling long distances for heart valve surgery; and negotiating the transition from paediatric to adult care [28,30,31,34,36]. An RHD diagnosis affects the whole family. The impacts on children include: not being able to do what other children can; the pain and inconvenience of injections in the context of school and sports; missing out on parents who are travelling for care because of their RHD; and growing up without parents who die from RHD related causes. Finally, deaths in hospital may mean people are buried off country [36].

“*One of the biggest implications of her disease was that we were taken off country…because of her condition, we lived all over Australia at different missions and communities and, sadly, my mum is buried off country*.” *Adult child of RHD patient quoted in Wyber et al., 2018* [36]

Analysis of the 201 combined extracted quotes revealed an overwhelming sense of collective trauma around RHD experiences within Indigenous families. This was expressed as feelings such as fears about death; anger towards healthcare services [27,29]; sense of unfairness at poor treatment [27]; demeaning system failures [9,27,29,30]; sense of abandonment [27]; unworthiness and lack of value as a person [9,27,29,30], especially through experiences of institutional racism. (NB Theme analysed differently as it uses a different data source and therefore not able to make a ‘relative strength’ comparison with other themes).

“*When they put the mask on me, I was screaming. It was really hard to do it.*” *Child with RHD quoted in Wyber et al., 2018* [36]

“*The health centre, that time, they didn’t help me. I always took her for a check-up. At night time I always took her, and the health workers gave her Panadol and told her to come back in the morning for a check-up. And I told them she has got this shortness of breath, and they told me that the Panadol will help, and tomorrow she will get a check-up*.” *Parent of child with RHD quoted in Harrington, 2005* [27].

##### Experiences of Medications and Adherence 

Much of the reviewed literature focuses on the delivery of secondary prophylaxis injections, usually required for at least a decade. Thus, in this relatively well-developed body of literature there was a strong focus on service delivery, identifying barriers to adherence and program evaluations. 

The reviewed publications reflect a tension regarding who has ultimate responsibility for medication adherence [8,10,26,27,28,29,33]. This tension was often described in terms of blame (service provider attitudes) [27,29,34] and powerlessness (community perceptions) [9,25,30,31]. For example, there was a conflict between parents wanting their children to receive their injections and yet not wanting to inflict a painful and distressing procedure upon them against their wishes [10]. In one study, a preference for health care workers to actively seek out those requiring secondary prophylaxis to the point of delivering home care was preferential to parents taking their children to the clinic for injections [27]. This shifted the locus of responsibility to a healthcare provider and removed the parent’s sense of inflicting pain on their children while still ensuring care was provided for the child. Overall, responsibility was variously attributed to the health service (invoking the need for good recall systems, good connection with clients, timeliness and flexibility) [26,27,29,33] or parents (who are often considered responsible for their children’s welfare in all matters) and/or the patients themselves [10,29,34]. 

It was reported that health service providers associated non-compliance, particularly in younger children, with a parental failure to care for their families [10,27,29,30,34]. Clinicians did not perceive the lack of understanding of families to be a reflection on the mode of delivery of care or inadequate explanations of illness; but instead considered parental confusion to be a direct shortcoming in fulfilling their responsibility. Others suggested parents were uncaring about their children’s health. One publication provides an example of negative language used by healthcare providers with regard to compliance and illness.

“*Either they don’t understand, or they don’t want to listen, and that’s their prerogative really. They expect us to create miracles and keep them alive, yet they’re not doing their part of the bargain*” *Healthcare provider quoted in Read et al., 2018* [34]

In interactions with the healthcare system parents interpreted ‘victim blaming’ messages from health professionals as cited above, who implied parents’ circumstances were directly under their control and not influenced by external, unpredictable factors [9,10,26,27,35,36]. The New Zealand literature adds depth to this, describing families’ experiences around expectations that they could ensure children’s adherence to prophylaxis without exception [39] and another described parental remorse at not realising the extent of their child’s illness, and not seeking care earlier [41].

Conversely, noncompliance was not perceived as a problem by some Aboriginal people [27,32,35] and in fact may sometimes be observed as a powerful act “*without power to be heard or to negotiate, Aboriginal children and young people may use the only power they have, which is to refuse or avoid injections*” [32].

##### Experiences of Pain Associated with Medication 

The mainstay of management for ARF remains the long term and regular regimen of penicillin injections which makes the issue of injection pain unavoidable, and for many children and their families, a central issue. A subset of papers explored pain associated with injection delivery, generally in the context of evaluating barriers to adherence [10,26,27,32,36].

*“The hardest part of living with rheumatic heart [disease] is to keep having the injections. It makes me feel really sad and sometimes mad. It’s really, really, hard,” 5-year old female with RHD quoted in Wyber, et al., 2018* [36]

*“It hurts a lot. I just wanna find out when it stops”* and *“[injection pain] got worse and worse and I couldn’t walk” Child with RHD quoted in Mitchell et al., 2018* [32]

The fact that it is predominantly Aboriginal children requiring the injections, and the majority of healthcare providers giving the injections are white, thus representing the dominant and the privileged (non-Aboriginal), means the repeated painful episodes have potential to deepen already felt inferiority and lack of power among Aboriginal families [32].

Some healthcare providers spoke about experiencing vicarious trauma in the process of giving injections seen as ‘intrusive’, patronising, exploitative of the power differential, painful, ‘persistent’, unpredictable and chaotic [10,27,32]. In contrast, the clinical management of injection pain was found to be perfunctory and haphazard [10,28,29,30,32] or poorly informed, for example accepting patient stoicism as normal [30]. Injection pain experiences indicated that there were deficits in clinician’s skills to manage episodes competently through child-focussed care or adolescent care [10,31,32].

*“When I get the injection, it is painful for two to three days. I limp, and it sometimes keeps me awake at night. It leaves a lump in buttock that is painful to touch.” An 18-year-old female on secondary prophylaxis regimen for 7 years quoted in Mitchell et al., 2018* [32]

Several publications described insights from Indigenous adolescents on their experiences of pain, finding that there were variable experiences and fears associated with uncertainty around the injection regimen [10,30,31,32,33].

#### 4.1.3. Health Service Domain

In addition to the sociological factors and RHD disease specific factors discussed above, the reviewed publications described the impact of a range of health service-related factors. Dominant themes were inadequate access to primary healthcare and the impact of poor health communication. Finally, not all publications reported negative experiences, some described factors that can contribute to positive experiences and high-quality care delivery. These three themes are discussed in more detail below.

##### Inadequate Delivery of Healthcare

The literature commonly reflected that access to healthcare was considered inadequate and culturally unsafe. Reviewed publications identified impediments to accessing health care, including: part-time availability of services; doubts about confidentiality; inappropriate gender of healthcare providers; questions of competence, and including that services did not meet needs [9,25,27,29,34]. The papers identified failure of services to take into account factors such as poverty, unemployment, powerlessness and social obligations [8,25,35,36] (identified in earlier themes as significantly impacting lived experience). The New Zealand literature described additional impediments to accessing essential primary care including the incidental costs of seeking care such as taking time off work to attend a General Practitioner appointment with a child, lack of transport to services or the cost of medications [30,37,42].

At a system level, insufficient resources and policy decisions result in limitations to healthcare provided to RHD patients, particularly in remote and regional areas [8,9,10,25,32]. Limitations include treating only the disease, as opposed to comprehensive care, and having a lack of understanding of the lived realities of hardship, sufferance and invisibility of the day to day struggles of Indigenous people [9,10,25,27,30]. One specific area of health service gap is for young people transitioning from paediatric to adult care (both at primary and tertiary service levels). Particularly, given inadequate expertise in Adolescent Health, this results in young people getting lost in the system with poor or no follow up due to lack of communication between health professionals [10,30,31,33].

##### Health Communication

While the reviewed literature recognised the need for patients to have a clear understanding of ARF and RHD, barriers to effective health communication were frequently identified. This was a significantly more cited theme than any other in our review (27 instances compared to 22 for the next most cited theme—Experiences of medications and adherence) and every publication discussed some aspect of communication.

Access to knowledge and information was discussed in most publications. Nearly all publications found that family members and patients self-identified a limited understanding of the disease, its causes and methods of prevention [8,9,10,26,27,28,29,30,31,33,34]. Many Aboriginal people are not proficient in reading or speaking English as it is often not their first language [8,9,25,26,27,28,30,31,33,35], and there is a sociocultural/linguistic disconnection between largely non-Aboriginal healthcare systems and Aboriginal people [9,25,29,31,33,34]. Poor communication compromised ongoing engagement with health care and was often attributed to health staff providing biomedical explanations of the disease, which had no functional or conceptual meaning for patients [9,10,31,34].

*“During research interviews with interpreters the women said there were no words for ‘RHD’ or ‘heart valves’ in Aboriginal languages. These biomedical disease descriptions were new concepts that were poorly explained in the health education materials offered to women.” Belton et al., 2018* [8]

The requirement for patients to access multiple health professionals and retell their stories numerous times, adds to communication difficulties, particularly in the context of dealing with different healthcare levels (primary, tertiary); disciplines (maternity, cardiac); and jurisdictions (government, non-government and across states) [8,9,26]. Additionally, RHD care is often not in the repertoire of healthcare providers, and they are therefore unprepared and problems appear intractable [8,9,27,28,29,30,33,34].

“*But in this area, with patients and doctor, the communication isn’t working. They don’t understand each other. Sometimes the patients get angry or upset.*” *Relative of child with RHD quoted in Harrington, 2005* [27]

People affected by ARF and RHD reported feeling bad/shame and powerless arising from limited understanding of the disease due to healthcare providers using complex medical explanations [9,26,27,31,34]. Patients and families often felt unable to question healthcare providers or say that they didn’t understand, and frequently indicated a strong, unmet desire for meaningful information and communication about ARF and RHD. Knowledge was viewed as a valuable commodity that would be of benefit to community and family [9,27,28,29,30,34]. The denial of Aboriginal people’s languages within health institutions was identified as a denial of identity and a demonstration of dominance [33] as languages are clan and country identity markers within localised populations [33]. One paper suggested that failure to communicate may result in suboptimal care where people *“not fluent in English, who do not understand medical terminology, and who are not forthcoming with questions, are all at risk of being neglected”* [29].

In contrast, some of the reviewed publications discussed examples of effective communication such as being empowered [10] and use of own language [32] and being at ease with the place and time [34]. Information also needs to be delivered in a manner familiar to the individual or group to ensure understanding and in a manner that avoids producing negative feelings [28]. The ideal way of sharing information was described;

*“We have our own ways of understanding illness and health. Only by using our own words, metaphors that are meaningful to us, and a communication style that is respectful, can we hear the messaging from health professionals. This means the health messages need to be made with us rather than for us.” Aboriginal participant quoted in in Haynes et al., 2019* [27]

##### Factors Contributing to Positive Experiences of Health-Care

Not all publications reported negative experiences, some described factors that can contribute to positive experiences and high-quality care delivery. Many of the reviewed publications noted that when families and/or healthcare providers were actively considerate of patients’ feelings and engaged well with families, treatment uptake increased [9,25,26,27,30].

*“‘Good care’ for patients with RHD was often discussed using the terms djäka, meaning to care for physically, and gungga’yun, translated as to encourage or to nurture.” Harrington et al. 2006* [28]

Families were viewed as important facilitators in enabling members to access care, be it via emotional support, material resources, or reminding people when their injections were due [8,9,26,27,28,30,33]. The role of the wider community in the provision of care was also important. Local community navigators who spoke local languages and understood cultural and community issues were viewed as essential collaborators with health service providers, complementing and enhancing service delivery through the relationship [27,28,31]. Community members in one study opposed an individualistic focus, preferring a whole group approach [28]. Health care provider roles included: engaging with families, offering ‘pastoral’ care; visiting people at home, talking to families, encouraging and caring for patients emotionally, like a family member (relational care) [10,25,26,27,28,29,30,32].

When providers acknowledged the wider community and the challenges contained within community living, they became more willing to engage with the complex lives of patients and families by undertaking non-health service activities, and delivering care more frequently at home, thus becoming an asset to those living with ARF and RHD [25].

### 4.2. Interacting Themes

Despite being described separately, the three theme domains (sociological, RHD disease specific and health service-related factors) are recognised as interacting synergistically. The realities of injection pain for children and adolescents demonstrates, for example, the overlapping theme domains impacting on the lived experience of RHD. That is, injection pain is associated with:

- Angst and uncertainty about who holds responsibility for the injections (Disease specific domain);
- Parents’ anguish seeing children in pain makes it difficult for them to assume responsibility for treatment, particularly when their child is unwilling to receive it (Disease specific domain);
- Lack of long term appropriate support and counselling for the regular painful procedure (Health service domain);
- Lack of power to communicate and negotiate with healthcare providers on managing their child’s injection pain (Sociological domain);
- Inadequate opportunities to gain health literacy to do with the injections in the context of trauma (Sociological domain). Additionally, associations between a variety of life experiences and difficulties can magnify trauma. For example, the stress of being hungry due to lack of household food is potentially magnified, through the trauma of having to attend the clinic to receive a painful injection.

### 4.3. Critical, Interpretive Analysis of Reviewed Publications

Despite using extensive search terms and broad inclusion criteria, only a small amount of relevant literature was identified to review, due to the lived experience of RHD not being part of the dominant medical discourse. Only three of the reviewed publications were purely interested in the Indigenous voice. Most were published in biomedical journals, had biomedically trained authors and were focused on solving biomedical issues, in particular compliance with long-term injections. Additionally, the biomedical literature related to RHD is dramatically larger; a recent Report (Endgame, *in press*) includes hundreds of biomedical references. The literature inclusive of any qualitative research is also a relatively more recent (10 of the 15 reviewed publications were published since 2016). This reflects the lack of focus and possibly limited incentives to investigate the lived experience of RHD relative to researching measurable prevention and treatment modalities. As a whole, biomedical science has only recently recognised the ethical imperative to include views of patients in research or health service planning and correspondingly there is a lack of attention to sociological complexities. In addition, given that the reviewed literature was mostly published in biomedical journals, even when authors had conducted sound qualitative lived experience research, reported findings tended to be limited due to journal requirements (see for example Read et al. [34]).

#### 4.3.1. Impact of Biomedical Focus in the Reviewed Literature

The impact of the paucity of qualitative research and the strong biomedical focus in the dominant narratives regarding RHD is evidenced in the limited nature of some of the themes identified in the review. Noteworthy omissions in the reviewed literature include: the experiences of children and adolescents; evidence of Indigenous priorities and perspectives related to policy and practice; nuanced discussions of power relations (for example regarding medication compliance); recognition of Indigenous knowledges and strengths as central; and a lack of critical reflection regarding the impacts of a dominant biomedical approach. These are discussed in more detail below.

#### 4.3.2. Knowledge Gap Regarding Children and Adolescents

The term ‘people’ was used throughout much of the reviewed literature (for example ‘people living with RHD’). However, the term ‘people’ tends to distract from the fact that it is children and their families that are predominantly affected. To think logically about prevention, it makes sense that the focus needs to be on children and families. Use of the term ‘people’ is mirrored by the large gaps in the reviewed literature relating to children’s and adolescents’ lived experience. Notable exceptions in the reviewed literature were the work of Mitchell et al. [30,32,33] and Spray et al. from the New Zealand literature [43]. We argue that a greater focus on children and adolescents would build on the Indigenous cultural strength that recognises the place of children in the continuity of Indigenous society; that is, they grow up to be adults with the responsibility to care for their culture and country; *“Children are our future”* [44]. This requires different resourcing and co-design of health systems in order to prioritise local community input and to recruit healthcare providers with training in Adolescent and Child Health.

#### 4.3.3. Indigenous Priorities and Perspectives (Related to Policy and Practice)

In the sociological domain themes, it was noted that there was a little discussion regarding factors conventionally thought to impact on RHD (e.g., health hardware, overcrowding). This reflects the extent to which Indigenous priorities are often not those of biomedical research. This was illustrated by a community-based action research project, where a shift in the original aims of the study was negotiated to areas the community saw as priorities *“which did not have such well recognised biological associations with RHD”* [28]. Further, an initial focus on individual ‘behaviour change’ was relinquished for an Indigenous collectivist framing of the research problem and areas for action. A similar dissonance can be observed between the dominant biomedical view of crowding as a primary cause of ARF/RHD [45] and, as has been noted elsewhere, the observation that the discourse of ‘overcrowding’ fails to recognise the cultural strengths of multigenerational close living [44,45,46]. Additionally, the review families who were described as having a large number of people in the house reported feeling judged or *“spied on”* by healthcare providers for not meeting ‘Western’ expectations of house standards [26]. The literature is silent on how this strength could be utilised in a positive way to approach and address the health issues that can emerge through close living in deficient or inappropriate housing.

Finally, given the recognition that Aboriginal Community Controlled Health Organisations (ACCHOs) can often better meet the needs of Indigenous peoples in a culturally safe context [47] it could be assumed that people living with RHD would benefit from the services provided by ACCHOs. However, none of the publications involving ACCHOs as study sites [27,33,34] provided any evidence regarding the quality of service, nor was there any indication that having Indigenous staff mitigated against poor experiences or improved knowledge [25,27]. This gap demonstrates a lack of attention to Indigenous priorities in biomedical research in the reviewed literature. Elsewhere, the medicalisation of health services has been identified as marginalising Aboriginal health workers and their traditional role [48] and contributing to the cultural incompetence of Western biomedicine [31,33].

#### 4.3.4. Noncompliance—Who Is to Blame and Whose Behaviour Needs to Change?

Investigating barriers to the uptake of long-term injections (compliance/adherence) was a focus of nearly half the reviewed publications. A commonly reinforced view was that noncompliance was a failure of families to provide care and a perceived parental confusion was attributed to a direct shortcoming of responsibility. The language of noncompliance is bound up with medical authority and control and may leave entrenched institutional arrangements unquestioned [49] thus contributing to asymmetric power relationships between staff and patient. The privileging of biomedicine may result in a *“pervasive communication failure”* [50] where the health system fails to provide accessible, acceptable, and/or effective services for Aboriginal clients, while then laying the *“blame for ‘non-compliance on the Indigenous clients, establishing a cycle of suspicion”* [50].

It has been suggested that a healthcare focus on *“exhorting individuals to change their behavior at the expense of focusing on features of the environmental, political, or economic systems that produce ill health and inequity”* [51]. That is, focusing on behaviour change is seductively easier than focusing on broad causes of disease states.

#### 4.3.5. Recognition of the Centrality of Indigenous Knowledges and Strengths

In nearly half of the reviewed publications there were descriptions of protective factors associated with Indigenous ways of being and doing. As stated previously, relationship-based strengths are gained from being part of a collective group that is culturally mandated and connected to local knowledge/languages and specific parts of country. However, these strengths were often not identified as such in the reviewed publications. This reflects the influence of a Western biomedical individualistic concept of health *“discernible at the heart of much health policy”* [51]. Communication issues are frequently discussed in the biomedical literature but are mostly considered in the context of an individualistic model of clinical care (i.e., a problem related to clinician-patient interactions only) [51]. This fails to recognise the communitarian basis of conceptual knowledge.

#### 4.3.6. Critical Thinking Regarding Biomedical Dominance

Biomedicine as a discourse became dominant in the 20th century *“and insists that all disease has an identifiable biological cause and can be treated without taking social life into account; defines health as an absence of pathology; and emphasises curative medicine”* [52]. As a consequence, Western expertise has become a dominant and self-sustaining approach in health research. The above discussion of gaps in the reviewed literature illustrate how biomedicine can lead to blind spots, predisposing researchers, policy makers and service providers to uncritically accept information or not ask how things might be different.

For example, the reviewed publications presented a relatively uncritical association between poverty/deprivation and RHD. The lack of research and interventions aimed at unravelling the mechanisms of poverty is at odds with the widespread acceptance of ARF and RHD as diseases of poverty [45], masking the complexities, trauma and practices of life, the neglect of which *“leaves the root causes unchanged”* [45]. This view tends to ignore the lived realities, that is, the social, cultural, economic, historical factors that contribute to how life is experienced.

The impact of a focus on biomedical knowledge and, more broadly, Western scientific knowledge, as a superior and more valid form of knowledge, is seen by critical Indigenous theorists as part of a coloniser’s perspective that perpetuates Othering and systematic racism [16]. Thus, dominant medical narratives and interactions can be seen as mechanisms by which *“ideas about ethnicity and race are created, negotiated and resisted”* [35]. Othering as explained earlier, perpetuates a separateness, thus giving continued power to the dominant while invalidating Indigenous knowledges.

### 4.4. Relative Strengths of Themes

Using the hierarchy of evidence to rank publications from this review, as well as counting the most frequently cited themes, provides guidance regarding the evidence that most needs to be taken into account in practice, policy and research. Some themes were more highly cited, either generally by all publications, or specifically by publications considered to be more highly ranked according to the hierarchy of evidence [22]. By either of these measures, ‘Health communication’ (containing sub-themes related to the causes and impacts of poor communication) was a dominant theme. Other dominant themes were ‘Experiences of medication and adherence’; ‘Experiences of pain’; and ‘Experiences of power differences and racism’. Factors contributing to positive experiences of healthcare (including the sub-themes ‘Being considerate of feelings’ and the ‘Roles of families and communities’) was notable as a dominant theme that reflected Indigenous cultural strengths (see Table 2).

Power relations are the common thread in these dominant themes. Furthermore, in the sociolinguistic analysis of participant quotes from all reviewed publications, trauma relating to the impacts of colonisation (loss of lands, livelihoods and non-recognition of identity, dis-empowerment of racism) and feelings related to lack of power/agency were identified as dominant themes. Trauma as a determinant of health experiences provides an alternate narrative that requires further exploration (Mitchell, *forthcoming*).

## 5. Discussion

As the review findings demonstrate, RHD is a complex disease, primarily affecting children, adolescents and families who live in difficult circumstances and experience a high degree of powerlessness and marginalisation. The lived realities of sociological, disease and health systems factors intersect, producing a continuum of experiences, ranging from traumatic to supportive. Recognising these interactions necessitates creating change at every level of experience, addressing sociological as well as disease-specific and health system factors.

While the highly cited and/or highly ranked themes related to power differences provide a motivation for change, it is the themes that recognise Indigenous strengths that provide guidance regarding what needs to change. That is, the social practices described in the themes related to empathy/emotional care (being considerate of feelings) and ongoing collective relationships (families and communities), draw attention to the reasons why current individualised biomedical approaches have failed in this setting. As with other similar diseases, such as tuberculosis, biomedical ‘technical’ responses do not reflect the social nature of RHD [53]. In particular, the domains of Indigenous strengths (history, language, culture, knowledge) identified in the review recognise and reflect specific local realities and social contexts.

Changing health care delivery and empowering Indigenous people will require changes that can cause discomfort as a result of shifting the current hierarchies of power and disrupting the dominant white privilege norm. This will require implementing findings regarding the need for culturally responsive care based on analysis of the culture of the healthcare system and healthcare practitioners themselves [24]. Change will never be possible without discussions about power and inequity [54].

Without the capacity to radically shift macro level/income redistribution policies, describing RHD as “a disease of poverty” leaves little hope of creating change. Therefore, we propose that describing RHD as a disease of disenfranchised and marginalised peoples/communities offers better prospects to think about a way forward. Focusing on a human rights approach related to social justice/inequity has a greater potential to transcend politics although it is recognised that political will is important [55]. That is, focus can be shifted to questions such as what are the mechanisms that change social inequity at a local community level, and how might understanding and developing empowerment make a difference. These questions are best answered by broadening the disciplinary lens of research to include social science research that uses decolonising methods [43].

## 6. Conclusions

To improve care for children, young people, families and communities living with RHD, addressing the results of this review is critical, in particular focusing on healthcare service design and operation. Such improvements will require shifting the focus of healthcare, authority and control so that services are flexible, culturally safe, adaptive to local contexts and are family/community-based, as expressed by people living with ARF and RHD.

Providing good quality care requires health service providers to overcome the issues of inequity, cultural inappropriateness, complexity, and institutional racism. Consequently, a decolonising approach to primary health care *“is needed in order to improve shared decision making and alleviate power imbalances between healthcare providers and Aboriginal patients”* [30]. This requires changes to standard biomedical health service design and delivery. In particular, community-led, community co-designed research and action is needed. The collective trauma revealed by analysing participant voices points to the need for recognition of ongoing trauma experiences and for this to be addressed in care models for families. A reconfiguration of authority and power relations is required so that Indigenous knowledges and conceptions of health are prioritised, and Indigenous communities have the skills, capacity and opportunities to participate in and control how RHD is to be prevented and managed [53]. Privileging a biomedical approach, rather than seeing biomedicine as only one approach working alongside other disciplines, is to perpetuate the colonising of Indigenous health and continue the high rates of health disparity in the Indigenous population.

## Figures and Tables

**Table 1:** A summary of the Australian literature included in this review.

*Author, Date [Reference Number]*	*Presence of Indigenous Voice*	*Discipline of Authors*	*Type of Publication*	*Context-Urban, Rural, Remote*	*Evaluates an Intervention, Project or Program?*	*RHD ‘Journey Focus*	*Qualitative Appraisal*
Belton et al. 2016 [9]	Yes	Biomedical	Peer-reviewed publication	Remote NT	No	RHD during pregnancy	3
Belton et al. 2018 [8]	Yes	Biomedical	Peer-reviewed publication	Urban and remote	No	RHD during pregnancy	2
Chamberlain-Salaun et al. 2016 [10]	No	Biomedical	Peer-reviewed publication	Remote	No	Secondary prevention (LAB injections)	3
Gruen et al. 2005 [25]	No	Biomedical	Peer-reviewed publication	Remote	No	Disease prevention	4
Harrington et al. 2006 [26]	Yes	Biomedical	Peer-reviewed publication	Remote	Yes	Disease prevention	2
Harrington, 2005 [27]	Yes	Biomedical	Thesis	Remote	No	Whole ARF/RHD journey	1
Haynes et al. 2019 [28]	Yes	Social Science	Peer-reviewed publication	Remote	Yes	Secondary prevention (LAB injections)	1
Mincham et al. 2003 [29]	No	Biomedical and social science	Peer-reviewed publication	Rural and remote	No	Whole ARF/RHD journey	1
Mitchell 2017 [30]	Yes	Social science	Thesis	Rural and remote	No	Experience of injections	1
Mitchell et al. 2018a [31]	Yes	Biomedical and Social Science	Peer-reviewed publication	Rural and remote	Yes	Transition to adult care for children	1
Mitchell et al. 2018b [32]	Yes	Biomedical and Social Science	Peer-reviewed publication	Rural and remote	No	Secondary prevention (LAB injections)	1
Mitchell et al. 2019 [33]	Yes	Biomedical and Social Science	Peer-reviewed publication	Rural and remote	Yes	Whole ARF/RHD journey	1
Read et al. 2018 [34]	Not stated	Biomedical and social science	Peer-reviewed publication	Rural and remote	Yes	Secondary prevention (LAB injections)	3
Saethre, 2007 [35]	No	Social Science	Peer-reviewed publication	Remote	No	N/A	3
Wyber et al. 2018 [36]	No	Biomedical	Report	N/A	N/A	N/A	4

**Table 2:** Themes analysed according to hierarchy of evidence for practice (Daly et al.) [22] and frequency.

THEME	Level 1 Refs [27,28,29,30,31,32,33]	Level 2 Refs [8,26]	Level 3 Refs [9,10,34]	Level 4 Refs [25,36]	Totals
	Citations *	Citations *	Citations *	Citations *	
**Sociological factors**					
Lived realities	4	6	4	5	19
Experiences of power differences and racism	10	2	6	3	21
Indigenous culture, knowledge and strengths	9	1	5	3	18
**Disease specific factors**					
Disease specific and traumatic impact of RHD (sociolinguistic analysis)	43 ^ǂ^	1	8	7	NA ^ǂ^
Experiences of medications and adherence	11	3	7	1	22
Experiences of pain	12	1	3	1	17
**Health Service domain**					
Inadequate delivery of health services	7	2	5	3	17
Health communication	14	3	9	1	27
Factors contributing to positive experiences of health-care	11	3	5	2	21

* Citations—number of times content related to the theme appears in the reviewed literature. Table A2 Provides references numbers according to sub-themes. ^ǂ^ The subtheme ‘Disease specific and traumatic impact of RHD’ is based on a sociolinguistic analysis (a unique methodology) and therefore should not be compared to the weightings given to the other themes.

## Appendix A

### Appendix A.1. Terms Used to Capture Qualitative Research as a Proxy Search Term for Lived Experience

#### Appendix A.1.1. Search String

TS=(“rheumatic fever” OR “rheumatic heart disease”) AND TS = (“Australia” OR “New Zealand”) AND TS = ( qualitative OR “thematic analys*” OR “discourse analys*” OR “content analys*” OR narrative* OR audio$record* OR interview* OR vignette* OR “focus group*” OR ethnolog* or ethnograph* OR “grounded theory” OR phenomenolog* OR Heidegger OR merleau-ponty OR Husserl OR “mixed method*” OR sociologic* OR anthropologic* OR “colloquial evidence” OR “field study*” OR social OR cultural OR socio$cultural OR “community value*” OR “community priorit*” OR behavio$r* OR concordance OR adherence OR *compliance OR “care-seeking” OR “decision making” OR “decision-making” OR “selfcare” OR “self-care” OR barrier* OR “health knowledge” OR “health belie*” OR “health understanding” OR “lay knowledge” OR “lay belie*” OR “lay understanding” OR “patient* insight” OR “patient comprehension” OR “patient* knowledge” OR “patient* understanding” OR “patient* view*” OR “patient* opinion*” OR “patient* perspective*” OR “patient* perception*” OR psycholog* OR attitude* OR self-report* OR self-concept* OR self-efficacy OR “self-report*” OR “self concept” OR “self efficacy” OR “sense of” OR self-perception OR “self perception” OR empath* OR moral OR spiritual* OR humanis* OR hope* OR “body image” OR “body-image” OR “life change” OR “life-change” OR trust OR anxiet* OR resilience OR psychosocial OR psycho-social OR “illness paradigm*” OR “information dissemination” OR “Social identity” OR lifeworld OR “quality of life” OR “patient account” OR emotion* OR feel* OR acceptance OR satisfaction OR perception OR “patient experience*” OR “lived experience” OR “illness experience*” OR “experience of illness” OR “experiencing illness” OR “experience of living” OR “disease experience*” OR “experience of disease” OR “experiencing disease” OR “carer* insight” OR “carer* comprehension” OR “carer* knowledge” OR “carer* understanding” OR “carer* view” OR “carer* opinion” OR “carer* perspective*” OR “carer* perception*” OR “parent* insight” OR “parent* comprehension” OR “parent* knowledge” OR “parent* understanding*” OR “parent* view*” OR “parent* opinion*” OR “parent* perspective*” OR “parent* perception*”)

AND (qualitative) OR (thematic analys*) OR (discourse analys*) OR (content analys*) OR (narrative*) OR (audio*record*) OR (interview*) OR (vignette*) OR (focus group*) OR ethnolog* or ethnograph* OR (grounded theory) OR phenomenolog* OR Heidegger OR (merleau-ponty) OR Husserl OR (mixed method*) OR sociologic* OR anthropologic* OR (colloquial evidence) OR (field study*) OR social OR cultural OR socio*cultural OR (community value*) OR (community priorit*) OR (behavio*r*) OR concordance OR adherence OR *compliance OR (care-seeking) OR (decision making) OR (decision-making) OR (selfcare) OR (self-care) OR barrier* OR (health knowledge) OR (health belie*) OR (health understanding) OR (lay knowledge) OR (lay belie*) OR (lay understanding) OR (patient* insight) OR (patient comprehension) OR (patient* knowledge) OR (patient* understanding) OR (patient* view*) OR (patient* opinion*) OR (patient* perspective*) OR (patient* perception*) OR psycholog* OR attitude* OR self-report* OR self-concept* OR self-efficacy OR (self report*) OR (self concept) OR (self efficacy) OR (sense of) OR self-perception OR (self perception) OR empath* OR moral OR spiritual* OR humanis* OR hope* OR (body image) OR (body-image) OR (life change) OR (life-change) OR trust OR anxiet* OR resilience OR psychosocial OR psycho-social OR (illness paradigm*) OR (information dissemination) OR (social identity) OR (life world) OR (quality of life) OR (patient account) OR emotion* OR feel* OR acceptance OR satisfaction OR perception OR (patient experience*) OR (lived experience) OR (illness experience*) OR (experience of illness) OR (experiencing illness) OR (experience of living) OR (disease experience*) OR (experience of disease) OR (experiencing disease) OR (carer* insight) OR (carer* comprehension) OR (carer* knowledge) OR (carer* understanding) OR (carer* view) OR (carer* opinion) OR (carer* perspective*) OR (carer* perception*) OR (parent* insight) OR (parent* comprehension) OR (parent* knowledge) OR (parent* understanding*) OR (parent* view*) OR (parent* opinion*) OR (parent* perspective*) OR (parent* perception*)

#### Appendix A.1.2. Google Search Strings

((rheumatic fever) OR (rheumatic heart disease)) AND ((Australia) OR (New Zealand))

### Appendix A.2. Hierarchy of Qualitative Research Evidence as Developed by Daly et al. 2007

**Table A1:** A summary of the hierarchy of evidence-for-practice in qualitative research [22].

Study Type	Features	Limitations	Evidence for Practice
Generalizable studies (level I)	Sampling focused by theory and the literature, extended as a result of analysis to capture diversity of experience. Analytic procedures comprehensive and clear. Located in the literature to assess relevance to other settings.	Main limitations are in reporting when the word length of articles does not allow a comprehensive account of complex procedures.	Clear indications for practice or policy may offer support for current practice, or critique with indicated directions for change.
Conceptual studies (level II)	Theoretical concepts guide sample selection, based on analysis of literature. May be limited to one group about which little is known or a number of important subgroups. Conceptual analysis recognizes diversity in participants’ views.	Theoretical concepts and minority or divergent views that emerge during analysis do not lead to further sampling. Categories for analysis may not be saturated.	Weaker designs identify the need for further research on other groups, or urge caution in practice. Well-developed studies can provide good evidence if residual uncertainties are clearly identified.
Descriptive studies (level III)	Sample selected to illustrate practical rather than theoretical issues. Record a range of illustrative quotes including themes from the accounts of “many,” “most,” or “some” study participants.	Do not report full range of responses. Sample not diversified to analyse how or why differences occur.	Demonstrate that a phenomenon exists in a defined group. Identify practice issues for further consideration.
Single case study (level IV)	Provides rich data on the views or experiences of one person. Can provide insights in unexplored contexts.	Does not analyse applicability to other contexts.	Alerts practitioners to the existence of an unusual phenomenon.

### Appendix A.3. Further Thematic Analysis

**Table A2:** Themes and sub-themes analysed according to frequency and hierarchy of evidence for practice (Daly et al.) [22].

THEME	Level 1		Level 2		Level 3		Level 4		Totals
	Citations *	Reference	Citations *	Reference	Citations *	Reference	Citations *	Reference	
**Sociological factors**									
***Lived realities***	**4**		**6**		**4**		**5**		**19**
*Poverty/deprivation*	2	[27,28]	1	[8]	1	[9]	1	[25]	5
*Lack of transport*	1	[29]	2	[8,26]	1	[9]	2	[25,36]	6
*Mismatch in perceptions of overcrowding*	1	[27]	2	[8,26]	-	*-*	1	[25]	4
*Intersection of inequities*	-	*-*	1	[8]	2	[9,35]	1	[25]	4
***Experiences of power differences and racism***	**10**		**2**		**6**		**3**		**21**
*Lived reality - poor experiences; access difficulties*	3	[28,32,33]	1	[8]	1	[10]	1	[25]	6
*Biomedical dominance*	4	[27,30,32,33]			4	[9,34,35]	1	[25]	9
*Powerlessness/Normalisation of illness*	3	[28,30,33]	1	[8]	1	[9]	1	[25]	6
***Indigenous culture, knowledge & strengths***	**9**		**1**		**5**		**3**		**18**
*Community cultural strength*	3	[27,28,30]			1	[9]	1	[25]	5
*Cultural strength widely recognised*	3	[27,28,30]	1	[26]	2	[9,35]	1	[25]	7
*Relationship/collective strength culturally mandated*	3	[27,28,30]			2	[9,35]	1	[25]	6
***Theme totals***	23		9		15		11		58
**Disease specific factors**									
***Disease specific & traumatic impact of RHD***	**43** **^ǂ^**		**1**		**8**		**7**		NA ^ǂ^
*RHD is a complex condition*	3	[28,30,31]	-	*-*	1	[34]	1	[36]	5
*RHD affects the whole family*	-	*-*	-		-		1	[36]	1
*Disease specific and traumatic impact of RHD* ^ǂ^	40	[27,29,30,31,33]	1	[26]	7	[9,10,35]	5	[36]	NA ^ǂ^
***Experiences of medications and adherence***	**11**		**3**		**7**		**1**		**22**
*Shifting locus of responsibility*	6	[27,28,29,30,31,33]	2	[8,26]	2	[10,34]	-	*-*	10
*Health provider attitude to low medication adherence*	3	[27,29,30]	-	*-*	2	[10,34]	--	*-*	5
*Family and individual perceptions of low mediation adherence*	2	[27,32]	1	[26]	3	[9,10,35]	1	[36]	7
***Experiences of pain associated with medication***	**12**		**1**		**3**		**1**		**17**
*Injection pain*	4	[27,31,32,33]	1	[26]	1	[10]	1	[36]	7
*Vicarious trauma*	2	[27,32]			1	[10]			3
*Clinical management of injection pain perfunctory and haphazard*	6	[28,29,31] [30,32,33]			1	[10]			7
**Health Service domain**									
***Inadequate delivery of health services***	**7**		**2**		**5**		**3**		**17**
*Impediments to accessing health services*	2	[27,29]	1	[8]	3	[9,34,35]	2	[25,36]	8
*Health system limitations*	5	[27,30,31,33]	1	[8]	2	[9,10]	1	[25]	9
***Health communication***	**14**		**3**		**9**		**1**		**27**
*Causes of poor communication*	6	[27,28,29,30,31,33]	2	[8,26]	4	[9,34,35]	1	[25]	13
*Impacts of poor communication*	6	[27,28,29,30,31,33]	1	[26]	3	[9,10,34]	-	*-*	10
*Effective communication*	2	[28,30,32]	-	*-*	2	[10,34]	-	*-*	4
***Factors contributing to positive experiences of health-care***	**11**		**3**		**5**		**2**		**21**
*Being considerate of feelings*	3	[27,28,30,32]	1	[26]	2	[10,34]			6
*The role of families*	5	[27,28,29,30,32]	2	[8,26]	3	[9,10,35]	1	[25]	11
*The role of community*	3	[27,28,31]	-	*-*	-	*-*	1	[25]	4
***Theme totals***	**32**		**8**		**19**		**6**		**65**

* Citations – number of times content related to the theme appears in the reviewed literature. ǂ The subtheme ‘Disease specific and traumatic impact of RHD’ is based on a sociolinguistic analysis (a unique methodology) and therefore should not be compared to the weightings given to the other subthemes. For this reason, we have also not given subtheme and theme totals in this section.
